# An international comparative analysis of public reimbursement of orphan drugs in Canadian provinces compared to European countries

**DOI:** 10.1186/s13023-022-02260-6

**Published:** 2022-03-04

**Authors:** Leanne Marie Ward, Alexandra Chambers, Emine Mechichi, Durhane Wong-Rieger, Craig Campbell

**Affiliations:** 1 Department of Pediatrics, University of Ottawa, Ottawa, ON Canada; 2 Novartis Pharmaceuticals Inc, Dorval, QC Canada; 3 Canadian Organization for Rare Disorders, Toronto, ON Canada; 4 Children’s Hospital, London Health Sciences Centre, London, ON Canada

**Keywords:** Rare disease, Orphan drugs, Reimbursement, Regulatory approval, Funding decisions, Patient access

## Abstract

**Background:**

The Canadian government has committed to developing a national strategy for drugs for rare diseases starting in 2022. Considering this announcement, we conducted a comparative analysis to examine patient access to therapies for rare disease in Canada relative to Europe and the U.S.

**Methods:**

Given its similarity to the Canadian health care system, we used Europe as the reference point to analyze all of the therapies with an orphan drug designation approved by the European Medicine Agency (EMA) from 1 January 2015 to 31 March 2020. We then contrasted access to these drugs in Canada (Health Canada) and the U.S. (Food and Drug Administration, FDA). We focused on: (1) the number of therapies for rare diseases entering the Canadian market; (2) the percentage of these therapies that are publicly available to Canadians; and (3) the timelines for patients to access these therapies in Canada.

**Results:**

Sixty-three approved therapies with an orphan drug designation from the EMA were identified. Fifty-three (84%) of these drugs had also been submitted to the FDA for approval, and 41 (65%) were submitted to Health Canada for approval. In Europe, Germany, Denmark, and the U.K. had the highest percentage of publicly reimbursed orphan drugs (84%, 70%, 68%, respectively). In comparison, Ontario (32%), Quebec (25%), and Alberta (25%) had the highest percentage of drugs reimbursed among the Canadian provinces. The shortest median duration (in months) from EMA approval to jurisdictional decision on reimbursement was in Austria (3.2), followed by Germany (4.1), and Finland (6.0). In Canada, the shortest median duration (in months) from regulatory approval to reimbursement was in British Columbia (17.3), Quebec (19.6) and Manitoba (19.6), while the longest duration was in P.E.I (38.5), followed by Nova Scotia (25.9), and Newfoundland (25.1).

**Conclusions:**

Our comparative analysis found that relative to the EU Canadians had less frequent and timely access to therapies for rare diseases. This highlights the need for a rare disease strategy in Canada that allows for clear identification and transparent tracking of the pathway for rare disease drugs, and ultimately optimizes the number of patients with access to these therapies.

**Supplementary Information:**

The online version contains supplementary material available at 10.1186/s13023-022-02260-6.

## Background

When considered as a whole, the prevalence of rare disease is about 4–6% worldwide, which equates to 260–450 million persons living with a rare disease at any given point in time [1]. Many therapies have been developed to treat and manage some rare diseases. Traditionally, large clinical trials are conducted to demonstrate the efficacy and safety of the therapy for a specific disease. The results of these trials then inform decisions by health care systems around the world. In Canada, health care is administered provincially, whereby each of Canada’s ten provinces is responsible for its own delivery of health care within the mandate of the Canada Health Act [2]. Each province is charged with optimizing the delivery of care to their constituents.

An inherent challenge with therapies for rare diseases is that it is logistically difficult to conduct large, robust clinical trials for several reasons, not the least of which is the small number of patients in any one region or country. When larger and methodologically traditional clinical trials cannot be conducted, health care systems have difficulty under the current evaluation paradigms in assessing the value of the therapy. Many countries have developed strategies to overcome the obstacles regarding access for patients to therapies for rare diseases [3]. These strategies include creating unique mechanisms for the review of rare disease therapies, creating special budgets for these interventions, and developing preferential ‘scoring’ of therapies for rare diseases compared to treatments for other conditions [4]. While this challenge to generate supporting evidence to integrate novel therapies into rare disease management strategies has been acknowledged in Canada [5], currently there are no mechanisms to clearly identify rare disease drugs as distinct within the Canadian regulatory framework, nor to support the drug access process that specifically targets therapies for rare diseases. Recently the Canadian federal government committed $1 billion (Canadian) to launch a National Strategy for Drugs for Rare Diseases, starting in 2022 [6]. As of May 2021, the federal consultations on the strategy have indicated a focus on therapies associated with high cost, high unmet need, and high uncertainty; however, the details on the governance, structure, and funding allocation remain unclear. Nonetheless, patients with rare diseases and the clinicians who treat them welcome the spirit behind the initiative.

**Fig. 1:**
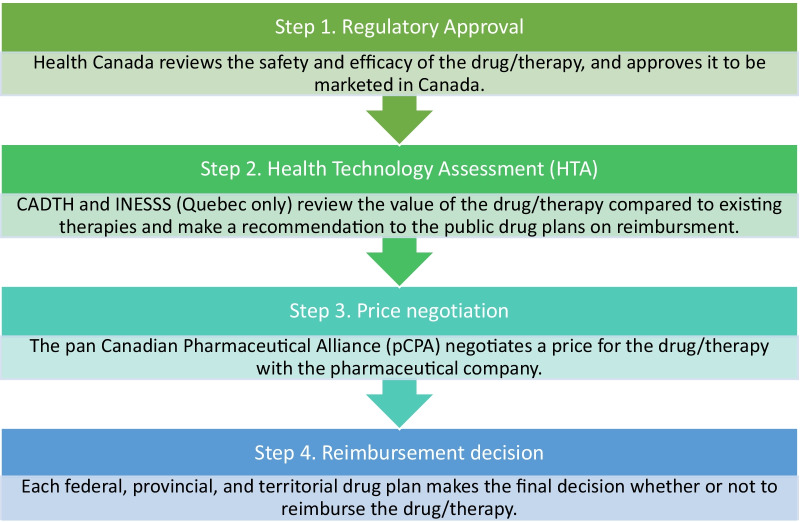
Drug Reimbursement Process in Canada. CADTH, Canadian Agency for Drugs and Technologies in Health; INESSS, Institut national d'excellence en santé et services sociaux

Many countries have complicated processes for reviewing drugs for public reimbursement, including additional steps when drugs for rare disease are being considered [4]. Not unlike other countries, Canada’s process for the public reimbursement of novel drugs is multi-step and complex (Fig. 1). All drugs entering the Canadian market first require regulatory approval by Health Canada; however, there are several steps after a drug receives Health Canada approval that are required before a drug is accessible to patients on a public formulary. Each step can take several months to complete, creating concern among clinicians and patients about ensuring timely access to therapies. After regulatory approval by Health Canada, in order for a drug to be considered for public listing on a provincial formulary, it must proceed through the Health Technology Assessment (HTA) pathway. Canada has two HTA agencies (Canadian Agency for Drugs and Technologies in Health [CADTH] and Institut National d'Excellence en Santé et Services Sociaux [INESSS, Quebec only]) that review the value of the drug compared to existing therapies and make recommendations to the public drug plans on whether the drug should be reimbursed. If a drug receives a positive recommendation from an HTA agency, the next step in the process is to negotiate a price with the pan Canadian Pharmaceutical Alliance (pCPA) that can be used across Canada’s public drug plans. The final step in the process is for each province to make its own funding decision for the drug under review, based on the province’s budget and other priorities. Access for public and private drug coverage varies in Canada and access varies significantly across private plans depending on the specific plan sponsor. Since most Canadian patients with rare diseases rely on public drug reimbursement (for example, as a catastrophic cost outside of the private plan), it is appropriate to focus on the public drug plans for the purposes of comparison with European public reimbursement. Thus, private coverage is beyond the scope of this analysis.

Unlike Health Canada, both the European Medicines Agency (EMA) and the U.S. Food and Drug Administration (FDA) have criteria for orphan drug designations. These regulatory agencies use slightly different definitions to establish orphan drug status; notably the FDA’s criteria are broader than the EMA’s (Table 1).

**Table 1:** Comparison of FDA and EMA criteria for orphan drug designation

FDA Criteria [10]	EMA Criteria [7]
Drugs (includes biologics) for the prevention, diagnosis, or treatment of diseases or conditions affecting fewer than 200,000 persons in the US OR	The drug must be intended for a disease that is life-threatening or chronically debilitating
	The prevalence of the condition must not be more than 5 in 10,000; AND
Drugs that will not be profitable within 7 years following approval by the FDA	The medicine must be of significant benefit to those affected by the condition

EMA, European Medicines Agency; FDA, Food and Drug Administration

In light of the commitment to a national strategy for drugs for rare diseases that endeavours to overcome these barriers in Canada, we undertook a novel analysis to understand how Canada compares to other countries on access for patients to therapies for rare diseases. We focused on three key variables: (1) the number of therapies for rare disease entering the Canadian market compared to the EMA and FDA; (2) the percentage of these therapies that are publicly available to Canadians; and (3) the timelines for access for patients to these therapies.

## Methods

### Establishing the number of therapies for rare diseases with regulatory approval in Canada compared to Europe

We generated a list of drugs with an orphan drug designation approved by the EMA from 1 January 2015 to 31 March 2020. We chose this time period because we did not want any impact of the COVID-19 pandemic to unduly influence the observations, and this timing aligns with the U.K.’s gradual transition from EMA to using their own agency, Medicines and Healthcare Products Regulatory Agency (MHRA), for regulatory approvals [7]. As a result, the approval decisions made by the EMA also applied to the U.K. in this time period. The term “patient access” was used to describe the time required for the necessary approvals to take place in order for the patient to receive the therapy.

For this comparative analysis, we had to first create a reference list of rare disease drugs on which to base our comparisons. Given that Canada does not have any way to determine a list of specific rare disease drugs, we could not identify a list of rare disease drugs moving through the regulatory pathway. Thus, we used the EMA’s criteria when creating the list of drugs, because of the similarity in the health care systems in Europe compared to Canada. In addition, both Health Canada and the Canadian Institutes of Health Research (CIHR) reference the EMA orphan drug definition in their documentation [8, 9]. For these reasons, we considered the EMA definition was more appropriate for the Canadian context than the FDA’s. We did include FDA in the comparison to be as complete as possible, recognizing some of the limitations of only comparing the EU with Canada. Using publicly available data from the FDA [10], EMA [11], and Health Canada [12], we established the status and dates of regulatory approval for the list of drugs that we generated using the EMA’s orphan drug designation. We also stratified the orphan drugs by the incidence of the rare disease and by cancer versus non-cancer indication. We used 1–5/10,000 versus < 1/10,000 for the stratification of incidence of rare disease because this aligns with the EMA’s definition for rare disease (5/10,000).

### Determining the percentage of the rare disease therapies that are publicly reimbursed in Canada

For the drugs with Health Canada approval, we determined the status and date of the HTA recommendation from both of Canada’s HTA agencies: CADTH and INESSS. If a drug received a positive recommendation from either HTA agency, we identified the date and status of the pan Canadian Pharmaceutical Alliance (pCPA) negotiations for a confidential drug price applicable to the Canadian public drug plans. And finally, if there was a pricing agreement reached on a pCPA negotiation for a drug, we searched the public websites of each of the provincial formularies to determine which provinces publicly reimbursed the drug. All data regarding HTA recommendations, pCPA negotiations, and provincial funding decisions were derived from the public data sources. The data sources used are listed in detail in Additional file 1.

### Benchmarking the timelines for patients to access therapies for rare diseases

Upon extraction of data from the various stages of the drug access process, we measured the timelines between each step of the drug access process and compared patient access to these therapies across the ten Canadian provinces. We excluded Canada’s three territories from the analysis because of their substantial reliance on federal drug program funding and their relatively small share of the Canadian population (the three territories combined account for less than 1% of Canada’s total population) [13].

### Comparing patient access in Canada to Europe

Public access for patients was assumed in each Canadian province when the therapy was publicly listed on the provincial ministry of health formulary website. Generally, we determined that a drug for a rare disease was reimbursed in the European countries if it had a recommended (or partially recommended) funding status, it was listed on a national reimbursement list, or it was noted to have case-by-case reimbursement. We determined a drug was not reimbursed if there was a negative recommendation, no decision, or a decision pending. Every jurisdiction (province, country) has a different health system for providing access for patients. To conduct the comparative analysis several assumptions were made regarding access to therapy in each jurisdiction. The assumptions and exceptions are detailed in Additional file 1.

We compared the timeline from regulatory approval until reimbursement in Europe and Canada. For the European countries we measured the median difference from the date of EMA approval to the date of reimbursement decision in each country. For the Canadian provinces we measured the median difference of the date of the Health Canada approval to the date of the reimbursement decision in each of the provinces.

## Results

### Number of therapies for rare diseases entering the Canadian market

Between 1 January 2015 and 31 March 2020, the EMA approved 63 drugs with an orphan drug designation (Table 2). An additional 3 drugs were granted orphan designation by the EMA, but ultimately did not receive EMA approval. Fifty-three (84%) of these drugs were submitted to the FDA for approval, and 41 (65%) were submitted to Health Canada for approval. Notably, the FDA approved 356 drugs with orphan designation in this same time period which can likely be attributed to the fact that the FDA criteria for orphan drug designation are broader than the EMA criteria (Table 1).

**Table 2:** Drugs with EMA orphan designation approved from Jan 2015 to Mar 2020

Generic name	Brand name	Indication	Estimated incidence of indication^1^
Asfotase Alfa	Strensiq	Hypophosphatasia	1–5/10,000
Avelumab	Bavencio	Merkel Cell Carcinoma	1–2/500,000
Axicabtagene Ciloleucel	Yescarta	Primary Mediastinal Large B-Cell Lymphoma, Large B-Cell Lymphoma	1–5/10,000
Budesonide	Jorveza	Eosinophilic Esophagitis	1–5/10,000
Burosumab	Crysvita	Hypophosphatemia (X-Linked)	1–9/1,000,000
Cannabidiol	Epidyolex	Seizures (Lennox-Gastaut Syndrome), Dravet Syndrome	1–5/10,000
Caplacizumab	Cablivi	Thrombotic Thrombocytopenic Purpura	1/77,000 (France, less prevalent globally)
cenegermin-bkbj	Oxervate	Neurotrophic keratitis	1–5/10,000
Cerliponase Alfa	Brineura	Neuronal Ceroid Lipofuscinosis Type 2	1/ > 50,000
Chlormethine	Ledaga	Cutaneous T-Cell Lymphoma	1–5/10,000
Coagulation Factor Ix	Idelvion	Haemophilia B	1–9/100,000
Coagulation Factor Ix	Alprolix	Haemophilia B	1–9/100,000
Coagulation Factor X	Coagadex	Factor X Deficiency	1–9/1,000,000
Cytarabine & Daunorubicin	Vyxeos	Acute Myeloid Leukemia	1–5/10,000
Daratumumab	Darzalex	Multiple Myeloma	1–5/10,000
Darvadstrocel	Alofisel	Crohn's Disease (Fistulising)	1–5/10,000
Dinutuximab Beta	Unituxin	Neuroblastoma	1–5/10,000
Eliglustat	Cerdelga	Gaucher Disease	1–9/100,000
Gallium (68 Ga) Edotreotide	Somakit Toc	Diagnostic Use ((Gastroenteropancreatic Neuroendocrine Tumours)	1–5/10,000
Gemtuzumab Ozogamicin	Mylotarg	Acute Myeloid Leukemia	1–5/10,000
Gilteritinib	Xospata	Acute Myeloid Leukemia (Flt3 +)	1–5/10,000
Givosiran	Givlaari	Acute hepatic porphyria	1–5/10,000
Glibenclamide	Amglidia	Neonatal Diabetes	1/300,000
Glycerol Phenylbutyrate	Ravicti	Urea Cycle Disorder	1–5/10,000
Idebenone	Raxone	Leber's Hereditary Optic Neuropathy	1–9/100,000
Inotersen	Tegsedi	Transthyretin Amyloidosis	1–5/10,000
Inotuzumab ozogamicin	Besponsa	Acute lymphoblastic leukemia	1–5/10,000
Isavuconazole	Cresemba	Invasive Aspergillosis and Mucormycosis	1–9/100,000
Ivacaftor & Lumacaftor	Orkambi	Cystic Fibrosis	1–9/100,000
Ivacaftor & Tezacaftor	Symdeko	Cystic Fibrosis	1–9/100,000
Ixazomib	Ninlaro	Multiple Myeloma	1–5/10,000
Lanadelumab	Takhzyro	Hereditary Angioedema	1–9/100,000
larotrectinib	Vitrakvi	cancers with NTRK fusion	1–9/100,000
Letermovir	Prevymis	Cytomegalovirus Infection	1–5/10,000
Limbal Stems Cells, Autologous	Holoclar	Limbal Stem Cell Deficiency	1–5/10,000
Lutetium (177Lu) Oxodotreotide	Lutathera	Neuroendocrine Tumors (Gastroenteropancreatic)	1–5/10,000
Mercaptamine	Cystadrops	Cystinosis	1–9/100,000
Metreleptin	Myalepta	Lipodystrophy	1–9/1,000,000
Mexiletine	Namuscla	Myotonia	1–9/100,000
Midostaurin	Rydapt	Acute Myeloid Leukemia	1–5/10,000
Midostaurin	Rydapt	Aggressive Systemic Mastocytosis	1–9/1,000,000
Migalastat	Galafold	Fabry Disease	1–5/10,000
Nintedanib	Ofev	Idiopathic Pulmonary Fibrosis	1–5/10,000
Niraparib	Zejula	Ovarian Cancer	1–5/10,000
Nusinersen	Spinraza	Spinal Muscular Atrophy	< 1/1,000,000
Obeticholic Acid	Ocaliva	Primary Biliary Cirrhosis	1–5/10,000
Other Antineoplastic Agents	Zalmoxis	Haematological Malignancy (Haploidentical HSCT)	1–5/10,000
Panobinostat	Farydak	Multiple Myeloma	1–5/10,000
Parathyroid Hormone	Natpara	Hypoparathyroidism	< 1/1,000,000
Patisiran	Onpattro	Transthyretin Amyloidosis	1–5/10,000
Pegvaliase	Palynziq	Phenylketonuria	1/15,000
Pitolisant	Wakix	Narcolepsy	1–5/10,000
Polatuzumab Vedotin	Polivy	Diffuse Large B-Cell Lymphoma	1–5/10,000
Sebelipase Alfa	Kanuma	Lysosomal Acid Lipase Deficiency	1–9/100,000
Tagraxofusp	Elzonris	Blastic Plasmacytoid Dendritic Cell Neoplasm	1–5/10,000
Tasimelteon	Hetlioz	Non-24 h sleep–wake syndrome	< 1/1,000,000
Telotristat	Xermelo	Diarrhea (Carcinoid Syndrome)	1–9/100,000
Tisagenlecleucel	Kymriah	Large B-Cell Lymphoma	1–5/10,000
Transduced Cd34 + Cell	Strimvelis	Severe Combined Immunodeficiency (Adenosine Deaminase Deficiency)	1–9/1,000,000
Velmanase Alfa	Lamzede	Alpha Mannosidosis	1–9/1,000,000
Vestronidase Alfa	Mepsevii	Mucopolysaccharidosis VII	< 1/1,000,000
Volanesorsen	Waylivra	Familial Chylomicronemia Syndrome	1–9/1,000,000
Voretigene Neparvovec	Luxturna	Leber's Congenital Amaurosis (Rpe65)	1–9/100,000

^1^Source: https://www.orpha.net/consor/cgi-bin/Disease_Search_Simple.php?lng=EN

**Fig. 2:**
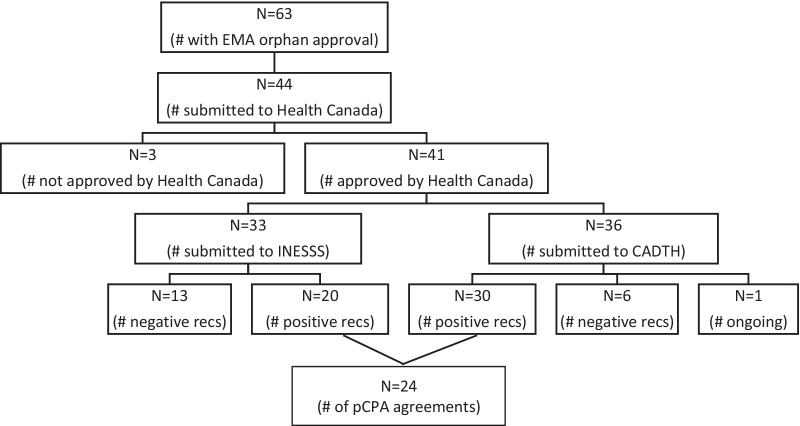
Flow diagram of drugs for rare diseases in the access pathway in Canada. CADTH, Canadian Agency for Drugs and Technologies in Health; INESSS, Institut National d'Excellence en Santé et Services Sociaux; recs, recommendations

After regulatory approval by Health Canada, a drug is submitted for an HTA review to establish if there is value for the drug compared to the existing therapies for the same indication. We found that of the 41 drugs with Health Canada approval, 36/41 (88%) had undergone an HTA review by CADTH, and 33/41 (80%) drugs had undergone an HTA review by INESSS (Quebec only). Of CADTH’s 36 HTA reviews, positive recommendations were issued for 30/36 (83%) and negative recommendations for 6/36 (17%), with one review ongoing as of 21 May 2021. Of INESSS’s 33 HTA reviews, positive recommendations were issued for 20/33 (61%) and negative recommendations for 13/33 (39%) reviews. In eight of 33 reviews, INESSS and CADTH made different recommendations, whereby INESSS issued negative recommendations for all eight drug reviews and CADTH’s issued positive recommendations for the same therapies (Fig. 2).

We found that 24 drugs had an agreement with the pCPA on a confidential price for the drug when the status of the drugs at the price negotiation step with the pCPA was reviewed. In addition, there were seven ongoing negotiations as of 21 May 2021. There was one instance where, despite a positive CADTH recommendation, a pricing agreement was not reached with pCPA (Eliglustat (Cerdelga) for Gaucher Disease). There was an active negotiation with pCPA where both CADTH and INESSS issued a negative recommendation, yet pCPA was still pursuing a pricing negotiation (Ivacaftor & Lumacaftor (Orkambi) for cystic fibrosis).

**Fig. 3:**
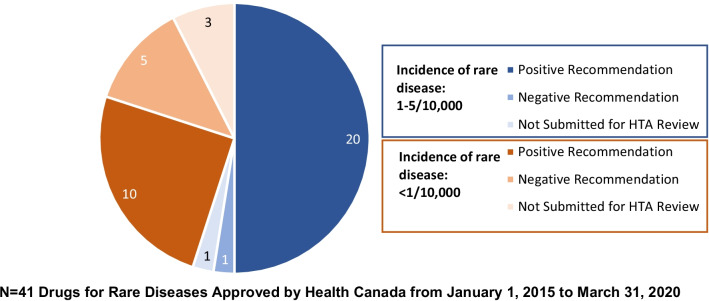
HTA Recommendation* Stratified by Incidence of Rare Disease. *This analysis was conducted for the CADTH HTA recommendations

We stratified the EMA approved drugs by the incidence of the rare disease (1–5/10,000 or < 1/10,000). There was a nearly even split between the 2 groups: 33/63 (52%) of the drugs had EMA approval for rare diseases with an incidence of 1–5/10,000, and 30/63 (48%) had EMA approval for rare diseases with an incidence of < 1/10,000. Similar proportions of the drugs also received Health Canada approval: 23/41 (56%) of the drugs had approval for rare diseases with an incidence of 1–5/10,000 compared to 18/41 (44%) for rare diseases with an incidence of < 1/10,000. There was a more notable difference with HTA recommendations, where 20/23 (87%) of the drugs for rare diseases with an incidence of 1–5/10,000 received a positive HTA recommendation (CADTH) compared to 10/18 (56%) for drugs for rare diseases with an incidence of < 1/10,000 (Fig. 3).

**Fig. 4:**
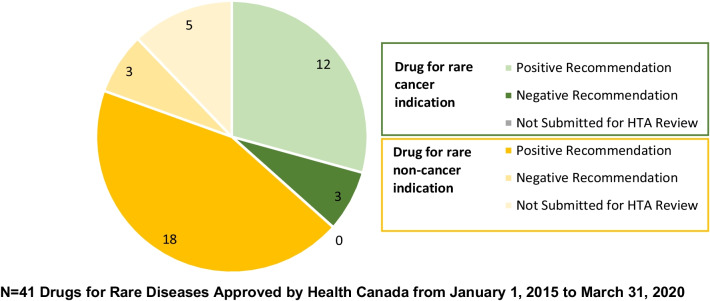
HTA Recommendation* Stratified by Cancer versus Non-cancer Indication. *This analysis was conducted for the CADTH HTA recommendations

We also stratified the EMA approved drugs by cancer versus non-cancer indication. Of the 63 drugs approved by the EMA, 20/63 (32%) were for cancer indications and 43/63 (68%) were for non-cancer indications. Proportionally more drugs for cancer indications were submitted for Health Canada approval: 15/20 (75%) compared to 26/43 (60%) drugs for non-cancer indications. Twelve of the 15 drugs for cancer indications with Health Canada approval received a positive HTA recommendation from CADTH compared to 18/26 (69%) of the drugs for non-cancer indications (Fig. 4).

### Proportion of rare disease therapies publicly available to Canadians

**Fig. 5:**
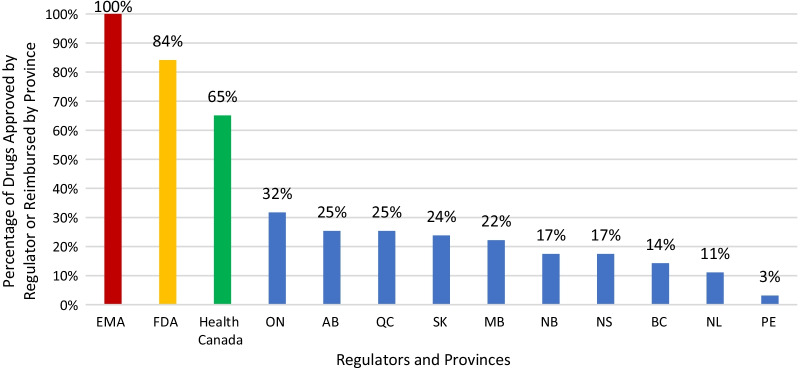
Percentage of drugs for rare diseases publicly funded by province compared to regulators. AB, Alberta; BC, British Columbia; MB, Manitoba; NB, New Brunswick; NL, Newfoundland; NS, Nova Scotia; ON, Ontario; PE, Prince Edward Island; QC, Quebec; SK, Saskatchewan

**Fig. 6:**
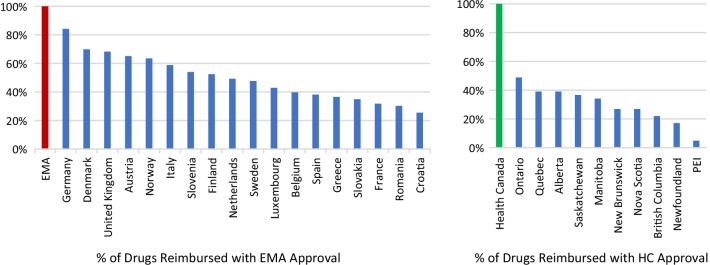
Proportion of orphan drugs with public reimbursement in European countries with EMA approval compared to provinces with Health Canada approval. EMA, European Medicine Agency; HC, Health Canada; PEI, Prince Edward Island

Since each province makes its own public funding decisions, there was variation observed across the country in terms of access to therapies for rare diseases. When we reviewed the list of 63 EMA-approved orphan designated drugs, we found that less than one-third of these drugs were publicly funded. Ontario had the most public patient access of the provinces with 20/63 (32%) of the drugs publicly reimbursed, followed by Quebec (16/63, 25%), and Alberta (16/63, 25%). On the other hand, Newfoundland (7/63, 11%) and Prince Edward Island (PEI) (2/63, 3%) had the least publicly funded access to these therapies (Fig. 5). We also compared the percentage of drugs publicly reimbursed in European countries by EMA approval to public reimbursement in each province by Health Canada and found that the proportion of public reimbursement for orphan drugs in Europe was generally higher than for Health Canada approved drugs in Canadian provinces (Fig. 6).

**Fig. 7:**
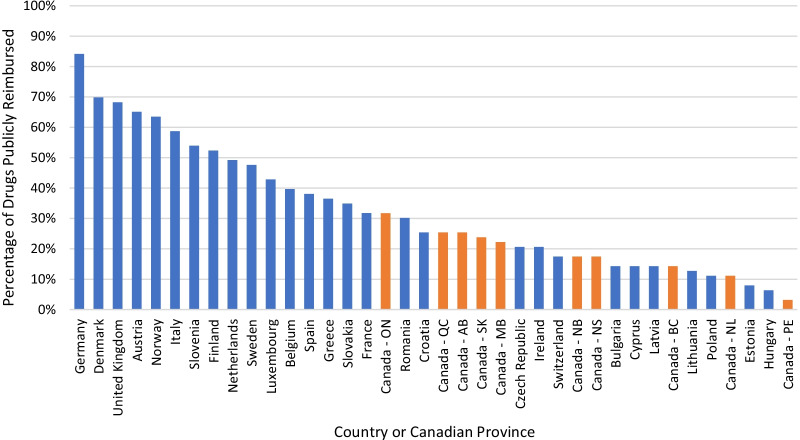
Percentage of therapies for rare disease that are publicly reimbursed. AB, Alberta; BC, British Columbia; MB, Manitoba; NB, New Brunswick; NL, Newfoundland; NS, Nova Scotia; ON, Ontario; PE, Prince Edward Island; QC, Quebec; SK, Saskatchewan

To provide additional context, the percentage of therapies for rare disease approved by the EMA that were available to patients in 27 European countries were compared to the data from the Canadian provinces (Fig. 7). We found that Germany had the highest access to the therapies for rare diseases approved by the EMA (84%), followed by Denmark (70%) and the U.K. (68%).

### Timelines for Canadian patients to receive access to therapies

Of the 41 drugs with Health Canada approval that fell within the 63 drugs with EMA orphan drug designation, we found that 7/41 (17%) of these drugs were approved by Health Canada before the EMA. When we compared the difference in regulatory approval timelines between EMA, FDA, and Health Canada, we observed closer timelines between the EMA and FDA, compared to Health Canada and EMA or Health Canada and FDA. The median lag time for EMA approval after FDA approval was 6.1 months compared to the median lag time of 11.0 months for Health Canada after FDA approval. There was substantial discrepancy in the median lag time for Health Canada to approve the drug after EMA approval, ranging from − 4.1 months (i.e., Health Canada approval occurred before EMA approval) to 41.2 months.

**Fig. 8:**
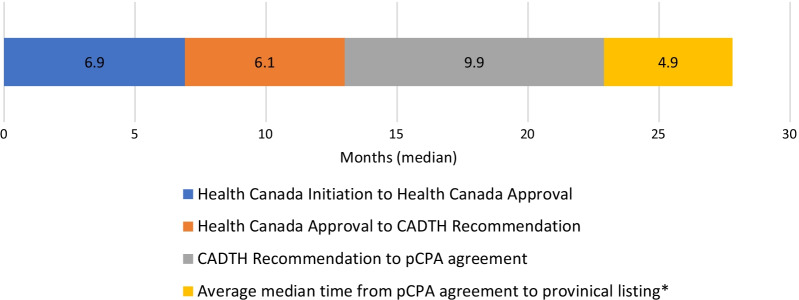
Median duration between steps in the Canadian drug access pathway. *PEI was excluded from the average provincial median timeline calculation because the timeline for PEI was only based on 2 drugs being reimbursed

Each step in the Canadian drug access pathway from Health Canada approval to a pCPA (price negotiation) agreement takes approximately 5–10 months (Fig. 8). The median time from Health Canada approval to pCPA agreement was 14.8 months (range 6.5–34.2 months). Overall, we observed the median time from the CADTH HTA recommendation to completion with pCPA was 9.9 months (range 1.6–24.8 months), and 8.2 months (range − 7.6 to 19.3 months) from the INESSS recommendation to a pCPA agreement.

**Fig. 9:**
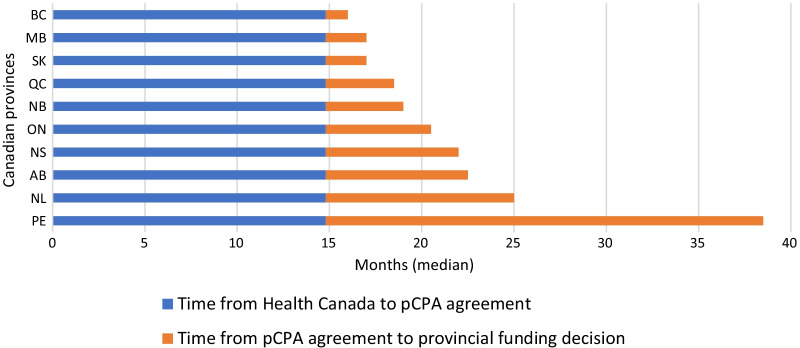
Median time (months) between Health Canada approval and public reimbursement by province. AB, Alberta; BC, British Columbia; MB, Manitoba; NB, New Brunswick; NL, Newfoundland; NS, Nova Scotia; ON, Ontario; PE, Prince Edward Island; QC, Quebec; SK, Saskatchewan

We found disparity in the timeline from Health Canada approval to provincial reimbursement. This is notable because all provinces would have followed the same timeline to the point of a pCPA agreement (median 14.8 months). After pCPA agreement (median 14.8 months after submission), provincial funding decision timelines vary between a median of 1.2 months (British Columbia) and 23. 7 months (PEI; Fig. 9). It should be noted, however, that there were only two drugs identified that were publicly reimbursed in PEI, so the sample was very small.

When comparing the timelines for the drugs by the incidence of rare disease or by indication (cancer versus non-cancer), there do not appear to be any notable outliers in terms of the median time in each step of the drug access pathway. Drugs for rare indications with the lowest incidence (< 1/10,000) appear to have a slightly longer time in Health Canada review than drugs for indications with an incidence of 1–5/10,000, however, the timeline from HTA recommendation to pCPA agreement appears slightly longer for drugs with an incidence of 1–5/10,000 (Table 3).

**Table 3:** Median time in months (range) between submission to Health Canada and pCPA Agreement

Step in pathway	All drugs with health Canada approval (n = 41)	Drugs for cancer indications (n = 15)	Drugs for Non-cancer indications (n = 26)	Drugs for indications with an incidence of 1–5/10,000	Drugs for indications with an incidence of < 1/10,000
Health Canada Submission to Health Canada Approval (months, range)	6.9 (6.0–23.7)	8.3 (6.1–18.5)	6.8 (6.0–23.7)	6.5 (6.0–18.5)	8.4 (6.1–23.7)
Health Canada Approval to HTA (CADTH) Recommendation (months, range)	6.0 (1.1–20.0)	5.0 (3.1–18.0)	7.6 (1.5–20.1)	5.5 (1.5–15.2)	6.1 (1.1–20.1)
HTA (CADTH) Recommendation to pCPA Agreement (months, range)	9.8 (1.6–24.8)	9.9 (4.3–14.6)	9.6 (1.6–24.8)	10.2 (4.3–18.2)	8.0 (1.6–24.8)

### Timeline for patients to access therapies for rare disease compared to Europe

**Fig. 10:**
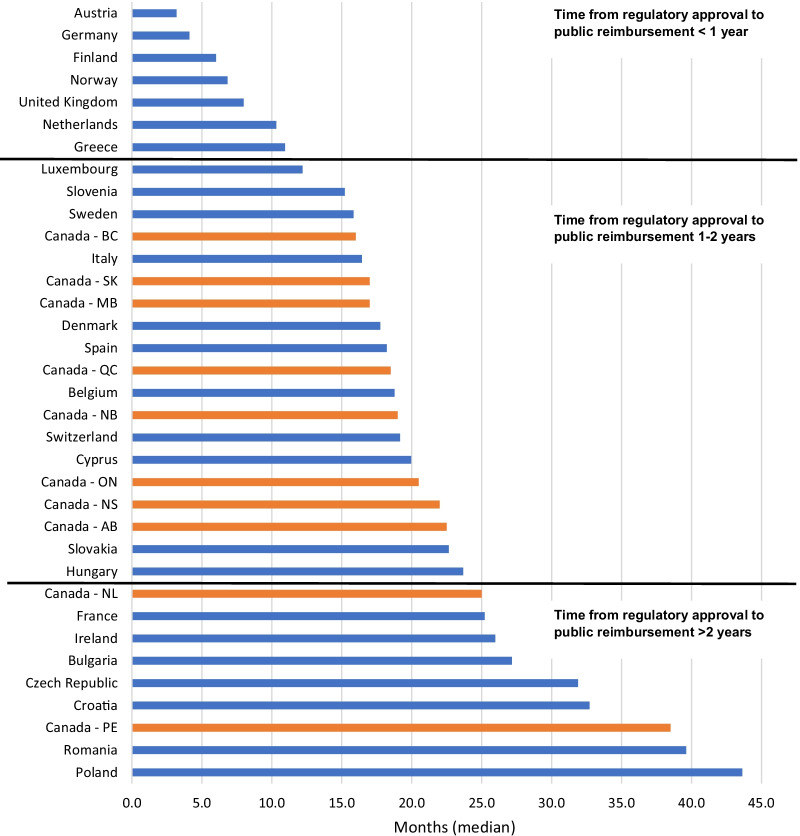
Timeline from regulatory approval (either EMA or Health Canada) and reimbursement. AB, Alberta; BC, British Columbia; MB, Manitoba; NB, New Brunswick; NL, Newfoundland; NS, Nova Scotia; ON, Ontario; PE, Prince Edward Island; QC, Quebec; SK, Saskatchewan

We compared the timeline from regulatory approval until reimbursement across 23 European countries to the Canadian provinces’ timeline for orphan designation drugs (Fig. 10). Austria had the shortest median timeline from regulatory approval to reimbursement decision (3.2 months), and Poland had the longest median timeline (43.6 months). The Canadian provinces had a median timeline of 1–2 years between Health Canada approval and public reimbursement, except for Newfoundland and PEI which both had median timelines beyond 2 years. It is important to use caution interpreting these latter two results since there were only two drugs reimbursed by PEI, and seven by Newfoundland, to inform the timeline calculations.

## Discussion

We undertook this analysis to compare access to therapies for rare diseases across Canada and in Europe because Canada is in the process of developing a National Strategy for Drugs for Rare Diseases [6]. This timely and novel analysis was important to understand how Canada fared by province, and in comparison to other countries. One of the factors that became evident early in our study planning is that, because Canada does not have a rare disease strategy, there is no way to determine a list of rare disease drugs moving through the Canadian regulatory approval process. Therefore, we used a list of rare disease drugs approved by the EMA with an orphan drug designation for our primary analysis. We chose the EMA as the reference because of the similarity of the Canadian health system to that of many European countries and the paradigm of public payer reimbursement being more congruent. However, we decided to include EMA approved drugs that had also been approved by the U.S. FDA as another frame of reference to understand the dynamics of rare disease drug approval and access. We did this because of the size, prominence, and close proximity of the U.S to Canada; and the interdigitation of the rare disease patient community. We recognize that there are many differences between the health systems in Canada and the U.S., and that FDA approval alone does not equate to drug access for all Americans.

We identified two key findings: (1) we found that of the therapies with an EMA orphan designation, fewer therapies for rare diseases were submitted to Health Canada than to the EMA or FDA for approval, and (2) that among those submitted to Health Canada, less than half were reimbursed on a public formulary in any province. Notably, we could not conduct the reverse analysis to measure orphan drugs approved by Health Canada, but not the EMA or FDA because Health Canada does not currently have criteria for orphan designation.

Other studies have demonstrated similar trends in which fewer drugs were submitted to Canada for regulatory approval compared with other countries in other disease areas, including cancer [14–16] and antibacterials [17]. The reasons for these discrepancies are not entirely clear, however it was suggested by Outterson et al. [17] that one possible explanation was the profitability for the pharmaceutical company in Canada was lower than in other jurisdictions. McKendrick et al. [16] noted that variation in availability to cancer drugs across countries was partially due to different willingness-to-pay thresholds. Similarly, Tunis et al. [18] identified a similar trend with cell and gene therapies, where fewer were available to patients in Canada and European countries compared to the United States, because of stricter decisions in the face of limited evidence. Tunis et al. [18] also noted that HTA agencies continue to heavily rely on evidence of higher quality studies (e.g., randomized controlled trials) in their assessments of value. This raises a particular challenge in the assessment of drugs for rare diseases, because it is not always possible to conduct robust trials in these populations.

### Disparity between Health Canada and EMA approvals

This comparative analysis has identified that fewer than two-thirds of the therapies approved by the EMA are even being submitted to Health Canada for regulatory approval. And while it is easy to blame the fact that Canada does not have a rare disease strategy in place, it is important to truly understand the component parts leading to this discrepancy. Could this be due to the small population of Canada relative to Europe, and that the resources required for a pharmaceutical company to bring their drug to Canada is not considered a good return on investment? Another possibility is the link between clinical trial activity in a country/region and the decision to bring to regulatory review. This was this was beyond the scope of our study but would be an important factor to examine in future work. Without further research, we cannot fully understand why there is the disparity in submissions to Health Canada compared with Europe. In 2019, Project Orbis was launched by the FDA to partner with other regulatory agencies, including Health Canada, with the aim of providing “patients faster access to promising cancer treatments across the globe” [19]. While Project Orbis is strictly focused on cancer therapies at this time, if proven successful, this program or similar models have the potential to be expanded to other therapeutic areas. Uncovering the factors leading to the rationale for the lower number of submissions made to Health Canada compared with the EMA will provide an opportunity to collectively strategize with clinicians, patient groups, federal and provincial governments, and the pharmaceutical industry on how to increase the number of drugs entering the Canadian market for approval.

### Low rate of public reimbursement of drugs with Health Canada approval

Beyond the issue of the number of therapies entering the Canadian market, there is the concern of the low number of therapies that successfully navigate the Canadian drug access pathway from Health Canada approval to public reimbursement on a provincial formulary. Half of the therapies submitted to Health Canada for approval proceeded to the point of a successful price negotiation with the pCPA, and then an even smaller proportion were found to be publicly funded in each province. This imbalance in access across provinces raises genuine equity concerns that warrant further exploration. An important subsequent research question would be to further understand why only half of the drugs progressed to this step and to explore uncertainty in the clinical evidence, comparable therapies in the same indication, and the costs of these therapies as possible hypotheses. It is also important to understand the role that patient advocacy groups and real-world evidence play in supporting the drug approval processes. If we can understand the positive and negative pressure points in the pathway, there is an opportunity to develop solutions or mechanisms to increase the availability to effective therapies for patients in Canada.

The due diligence and rigour that are required to ensure that a therapy is effective, safe, and affordable within the health system are critical for the sustainability of the health system. At the same time, these standards must be balanced against the pressing need to provide adequate treatments for those with debilitating or even life-threatening illnesses, to provide access to therapy that could improve or save lives. This balance, which further depends on a sustainable, fair access funding program, emphasizes the importance of a rare disease strategy that provides optimal therapy for patients so that they, like those with common disorders such as cardiovascular disease, chronic respiratory diseases, and diabetes, have an equal opportunity to live their best lives.

For many years, there have been articles written about the need for real-world evidence [20, 21] and outcomes-based agreements [22, 23] to address issues of uncertainty of the clinical value of therapies. In 2014, the provincial Ministers of Health in Canada developed an Expensive Drug for Rare Disease working group to pilot a proposal for supplemental processes for complex/specialized drugs, that builds upon existing review processes with health partners [24, 25]. The work of the Expensive Drugs for Rare Disease working group has yet to be delivered publicly. Understandably there is a concern in overwhelming an already overburdened drug access pathway by introducing new processes that require more time and resources to implement, however there are opportunities to gain efficiency such as providing conditional access to drugs while gathering real-world evidence to reduce the uncertainty in the HTA review. The rare disease community is an ideal setting to explore some of these mechanisms because of the small patient populations and, typically, the unmet need for effective therapies for patients. The Canadian Organization for Rare Disorders (CORD) has been actively engaged in supporting a robust and comprehensive strategy to support patients with rare diseases for several years, including most recently hosting consultations with a wide array of stakeholders to support the National Strategy for Drugs for Rare Diseases in Canada [26, 27].

### Limitations

We acknowledge that our comparative analysis has several limitations. There were three potential analyses that we did not carry out because we did not have access to the data. First, this analysis was a one-way assessment looking at drugs that were given an orphan drug designation by the EMA and following those drugs through the reimbursement process in Canada. We could not do the reverse and seek out Health Canada-approved drugs for rare diseases that were not approved by the EMA because Health Canada does not have orphan drug criteria. Secondly, since the orphan drug designation process by the EMA requires manufacturers to request orphan status, we may have missed drugs that would have met the orphan designation criteria by the EMA, but the manufacturer of the drug did not seek orphan designation. Finally, we had access to only publicly available sources of information, and there may have been cases in which a province in Canada provided “non-publicly documented” unique access to a rare therapy beyond the exceptional access program (EAP). So, while we can report on case-by-case access through the EAP processes, this may not include all approvals and therefore this analysis might under-represent actual access numbers. Nevertheless, since Canada’s health care system is predicated upon equal access to all, a reasonable first step was to examine the progress of access to rare disease therapies in the public domain.

## Conclusions

Rare diseases impact many people globally, and we are likely to see a continued effort by the research community to develop more new therapies for the multitude of conditions that fall into the broad catchment of rare disease. Our comparative analysis has unveiled that Canadians have less access to therapies for rare disease than those in the highest-access countries. In order for Canada to improve its record, we need a coordinated national strategy that will enable patients with rare disease to equitably and sustainably receive timely access to the most effective therapies. Of note, there are considerable challenges to conducting such comparative research on rare disease, given that Canada does not have a rare or orphan drug designation. We are encouraged that the Canadian federal government recognizes the need to have a strategy for high-cost drugs for rare diseases and anticipate that this will lead to a comprehensive rare disease strategy [6]. In support of such a strategy, we recommend further analysis to understand the root causes of (1) the disparity in drugs for rare disease entering the Canadian market compared to the EU, and (2) the considerable proportion of Health Canada-approved therapies that are not publicly reimbursed. Once we can understand these root causes, we can use them to inform the National Strategy for Drugs for Rare Diseases in Canada, which would result in improved access for Canadians who would benefit most from emerging therapies.

## Supplementary Information

**Additional file 1.** Data sources.

## Data Availability

All data were extracted from public sources.

## Abbreviations

CADTH: Canadian Agency for Drugs and Technologies in Health
EMA: European Medicines Agency
EU: European Union
FDA: Food and Drug Administration
HTA: Health Technology Assessment
INESSS: Institut national d'excellence en santé et services sociaux
pCPA: Pan Canadian Pharmaceutical Alliance
