# Supplementary material for: An international comparative analysis of public reimbursement of orphan drugs in Canadian provinces compared to European countries
Source: Orphanet J Rare Dis. 2022 Mar 4;17:113. doi: 10.1186/s13023-022-02260-6 (PMC8895096; doi:10.1186/s13023-022-02260-6)
Supplement: Supplementary file 1 — Additional file 1. Data sources. [file 13023_2022_2260_MOESM1_ESM.docx]

Additional file 1

**Table A1. Sources of data used for the comparative analysis**

| **Agency** | **Data Source** |
| --- | --- |
| **Health Technology Assessment Agencies** | |
| Canadian Agency for Drugs and Technologies in Health (CADTH) | Drug Review Reimbursement Database (1)  https://www.cadth.ca/reimbursement-review-reports |
| Institut national d'excellence en santé et services sociaux (INESSS) | Drug Evaluations (2)  https://www.inesss.qc.ca/thematiques/medicaments/medicaments-evaluation-aux-fins-dinscription.html |
| Pan Canadian Pharmaceutical Alliance (pCPA) | Negotiation Status (3)  https://www.pcpacanada.ca/negotiations |
| **Provincial Formularies/Drug Lists** | |
| British Columbia (B.C.) | BC Ministry of Health Formulary (4)  https://www2.gov.bc.ca/gov/content/health/health-drug-coverage/pharmacare-for-bc-residents/what-we-cover/drug-coverage |
| Alberta | Alberta Drug Benefit List (5)  https://idbl.ab.bluecross.ca/idbl/load.do |
| Saskatchewan | Saskatchewan Formulary (6)  http://formulary.drugplan.ehealthsask.ca/SearchFormulary  Exception Drug Access Program List (7)  https://formulary.drugplan.ehealthsask.ca/PDFs/APPENDIXA.pdf |
| Manitoba | Manitoba Pharmacare Program Database (8)  https://web22.gov.mb.ca/eFormulary/  Exception Drug Status List (9)  https://www.gov.mb.ca/health/mdbif/docs/edsnotice.pdf |
| Ontario | Ontario Drug Benefit Database (10)  https://www.ontario.ca/page/check-medication-coverage/  Ontario Exceptional Access Program Drug List (11)  https://www.health.gov.on.ca/en/pro/programs/drugs/odbf/odbf_except_access.aspx |
| Québec | Quebec RAMQ, includes Exceptional Access List (Quebec Ministry of Health) (12)  https://www.ramq.gouv.qc.ca/fr/professionnels/pharmaciens/medicaments/Pages/liste-medicaments.aspx |
| New Brunswick | New Brunswick Drug Plans Formulary (13)  https://www2.gnb.ca/content/gnb/en/departments/health/MedicarePrescriptionDrugPlan/NBDrugPlan/ForHealthCareProfessionals/NewBrunswickDrugPlansFormulary.html |
| Nova Scotia | Nova Scotia Pharmacare, includes Exception Status List (14)  https://novascotia.ca/dhw/pharmacare/formulary.asp |
| Newfoundland & Labrador | Newfoundland and Labrador Prescription Drug Program (NLPDP) Database (15)  https://www.health.gov.nl.ca/health/prescription/newformulary.asp  Special Authorization Drug List (16)  https://www.gov.nl.ca/hcs/files/Criteria-Feb-2021.pdf |
| Prince Edward Island (P.E.I.) | PEI Pharmacare Formulary (17)  https://www.princeedwardisland.ca/en/information/health-pei/pei-pharmacare-formulary |

**Table A2.** **Assumptions used to determine availability of the therapy for rare disease**

| **Country/Province** | **Reimbursement Status** | **Timeline of reimbursed access** |
| --- | --- | --- |
| Austria | Medicine is available if it is included in the reimbursement system on Austrian pharmacies list. | Date of appearance in Austrian pharmacies list |
| Belgium | Medicine is available if it is on the reimbursement list, or funded through the hospital. | Date of appearance in INAMI |
| Bulgaria | Medicine is available if it is on the reimbursement list. | Date of publication in Positive drug list - NCPR register |
| Canada (all provinces) | Medicine is available if it is on the reimbursement list. | Date listed on website |
| Croatia | Medicine is available if it is on the reimbursement list. | Date of publication in CHIF, Croatian Health Insurance Fund |
| Cyprus | Medicine is available if it is on the official MOH price list. | Date of inclusion in official price list |
| Czech Republic | Medicine is available if it is on the reimbursement list. | Date of inclusion in official reimbursement list published by SUKL |
| Denmark | Medicine is available if has received public reimbursement by Danish Medical agency, No information (not available) for case-by-case individual reimbursements. | Date of inclusion in official reimbursement list |
| Finland | Medicine is available if it is on the Pharmaceuticals Pricing Board publication list. | Date of inclusion in PPB pricing and reimbursement list |
| France | Medicine is available if it is on the reimbursement list, or funded through the hospital. | HTA body decision date considered form Drug Context Matters Market Access Platform and France MOH website |
| Germany | Medicine is available if it received positive recommendation received from GBA, IQVIG. | HTA body decision date considered form Drug Context Matters Market Access Platform |
| Greece | Medicine is available if it is on the reimbursement list. | Date of inclusion in official price publication by Greece MOH |
| Hungary | Medicine is available if it is on the reimbursement list | Date of inclusion in official NHIF publication |
| Ireland | Medicine is available if it is on the reimbursement list. | Date of inclusion in Ireland monthly price update |
| Italy | Medicine is available if it is on the reimbursement list, or funded through the hospital. | HTA body decision date considered form DRG Context Matters Market Access Platform and AIFA website |
| Luxembourg | Medicine is available if available in Belgium - automatic reimbursement following Belgium and official price list. | Date of inclusion in official price publication, PriCentric Launch dates |
| Netherlands | Medicine is available if it is on the reimbursement list. | Date of inclusion in official reimbursement list of Netherlands MOH |
| Norway | Medicine is available if it is on the reimbursement list. | Date of inclusion in official reimbursement list of Norway MOH |
| Poland | Medicine is available if it is on the reimbursement list. | Date of inclusion in official reimbursement list of Poland MOH |
| Romania | Medicine is available if the PriCentric database shows as reimbursed; Latest information in official Romania MOH publication is missing. | Date of Launch in the PriCentric Database |
| Slovakia | Medicine is available if it is on the official price list. | Date of inclusion in Lists of officially determined prices - Slovakia MOH, PriCentric launch date |
| Slovenia | Medicine is available if it is on the Central Medicines Database. | Central Medicines Database, PriCentric Launch date |
| Spain | Medicine is available if it received positive recommendation received from Spain MOH. | HTA body decision date considered form Drug Context Matters Market Access Platform. |
| Sweden | Medicine is available if it is listed on TLV drug database. | Timelines according to TLV drug database "Prices Valid from" date |
| Switzerland | Medicine is available if it is on the official reimbursement list of Swiss MOH. | Date of inclusion in PriCentric database |
| U.K. | Medicine is available if it received positive recommendation received from NICE. | HTA body decision date considered form DRG Context Matters Market Access Platform, PriCentric Reimbursement date |

**References**

1. Reimbursement Review Reports [Internet]. Cadth.ca. 2015 [cited 2021 May 29]. Available from: https://www.cadth.ca/reimbursement-review-reports

2. INESSS [Internet]. Inesss.qc.ca. [cited 2021 May 29]. Available from: https://www.inesss.qc.ca/thematiques/medicaments/medicaments-evaluation-aux-fins-dinscription.html

3. Brand Name Drug Negotiations Status [Internet]. Pcpacanada.ca. [cited 2021 May 29]. Available from: https://www.pcpacanada.ca/negotiations

4. Ministry of Health. Drug Coverage [Internet]. Gov.bc.ca. [cited 2021 May 29]. Available from: https://www2.gov.bc.ca/gov/content/health/health-drug-coverage/pharmacare-for-bc-residents/what-we-cover/drug-coverage

5. Alberta health - drug benefit list [Internet]. Bluecross.ca. [cited 2021 May 29]. Available from: https://idbl.ab.bluecross.ca/idbl/load.do

6. Home Page - Online Formulary 2021 [Internet]. Ehealthsask.ca. [cited 2021 May 29]. Available from: http://formulary.drugplan.ehealthsask.ca/SearchFormulary

7. Exception Drug Access Program List [Internet]. Ehealthsask.ca [cited 2021 October 29]. Available from: https://formulary.drugplan.ehealthsask.ca/PDFs/APPENDIXA.pdf

8. Pharmacare Program [Internet]. Gov.mb.ca. [cited 2021 May 29]. Available from: https://web22.gov.mb.ca/eFormulary/

9. Exception Drug Status List [Internet]. Gov.mb.ca [cited 2021 October 29] Available from: https://www.gov.mb.ca/health/mdbif/docs/edsnotice.pdf

10. Check medication coverage [Internet]. Ontario.ca. [cited 2021 May 29]. Available from: https://www.ontario.ca/page/check-medication-coverage/

11. Government of Ontario, Ministry of Health, Care L-T. Exceptional Access Program - Formulary - health care professionals - MOHLTC. 2009 [cited 2021 May 29]; Available from: https://www.health.gov.on.ca/en/pro/programs/drugs/odbf/odbf_except_access.aspx

12. Médicaments [Internet]. Gouv.qc.ca. [cited 2021 May 29]. Available from: https://www.ramq.gouv.qc.ca/fr/professionnels/pharmaciens/medicaments/Pages/liste-medicaments.aspx

13. Government of New Brunswick, Canada. New Brunswick Drug Plans Formulary [Internet]. Gnb.ca. 2014 [cited 2021 May 29]. Available from: https://www2.gnb.ca/content/gnb/en/departments/health/MedicarePrescriptionDrugPlan/NBDrugPlan/ForHealthCareProfessionals/NewBrunswickDrugPlansFormulary.html

14. Novascotia.ca. [cited 2021 May 29]. Available from: https://novascotia.ca/dhw/pharmacare/formulary

15. Health and Community Services [Internet]. Gov.nl.ca. [cited 2021 May 29]. Available from: https://www.health.gov.nl.ca/health/prescription/newformulary.asp

16. Special Authorization Drug List [Internet]. Gov.nl.ca [cited 2021 October 29] Available from: https://www.gov.nl.ca/hcs/files/Criteria-Feb-2021.pdf

17. PEI Pharmacare Formulary [Internet]. Princeedwardisland.ca. 2019 [cited 2021 May 29]. Available from: https://www.princeedwardisland.ca/en/information/health-pei/pei-pharmacare-formulary
